# Sedation level variability as an indicator for mortality in mechanically ventilated critically ill patients: a propensity score-weighted cohort study

**DOI:** 10.3389/fmed.2026.1755288

**Published:** 2026-01-16

**Authors:** Shu-Fen Liao, Chun-Jen Huang, Ru-Ping Lee, Tsung-Ying Chen, Hao-Chin Wang

**Affiliations:** 1 Department of Medical Research, Wan Fang Hospital, Taipei Medical University, Taipei, Taiwan; 2 School of Public Health, College of Public Health, Taipei Medical University, Taipei, Taiwan; 3 Integrative Research Center for Critical Care, Wan Fang Hospital, Taipei Medical University, Taipei, Taiwan; 4 Department of Anesthesiology, Wan Fang Hospital, Taipei Medical University, Taipei, Taiwan; 5 Graduate Institute of Clinical Medicine, College of Medicine, Taipei Medical University, Taipei, Taiwan; 6 Department of Anesthesiology, School of Medicine, College of Medicine, Taipei Medical University, Taipei, Taiwan; 7 Institute of Medical Sciences, Tzu Chi University, Hualien, Taiwan; 8 Department of Anesthesiology, Hualien Tzu Chi Hospital, Buddhist Tzu Chi Medical Foundation, Hualien, Taiwan; 9 School of Medicine, Tzu Chi University, Hualien, Taiwan

**Keywords:** ICU outcome, mortality, propensity-score weighted cohort study, RASS score, sedation level variability

## Abstract

**Objective:**

To investigate the impact of low versus high sedation level variability (SLV), measured during the first 72-h intensive care unit (ICU) stay, on clinical outcomes in ventilated critically ill patients.

**Methods:**

Patients were identified from the Medical Information Mart for Intensive Care IV database from 2008 to 2019. The SLV was quantified by calculating the coefficient of variation (CV) using 18 time-series exponentially transformed Richmond Agitation-Sedation Scale (RASS) scores for each patient. Based on the median of the CV, patients were divided into the low and high SLV groups, which were subsequently balanced by a stabilized inverse probability of treatment weighting method.

**Results:**

Compared to the high SLV group (*n* = 1749), the low SLV group (*n* = 1759) had a higher risk of 28-day [aHR (95% CI), 1.57 (1.36, 1.81); *p* < 0.001] and 90-day ICU mortality [aHR (95% CI), 1.51 (1.32, 1.71); *p* < 0.001]. However, these two study groups had similar outcomes regarding prolonged mechanical ventilation on ICU Day 14 and ventilation-free days on ICU Day 21. The negative impact of the low SLV on mortality was evident only in the subgroup with < 60% of RASS scores within the target range, but not in the subgroup with ≥ 60%.

**Conclusion:**

A reduced SLV, measured during the first 72-h ICU stay, is associated with an increased risk of both 28-day and 90-day ICU mortality in ventilated critically ill patients. Patients with < 60% of RASS scores within the target range are especially vulnerable to this negative impact.

## 1 Introduction

Adequate sedation is a crucial component of care for critically ill patients requiring mechanical ventilation in the intensive care unit (ICU) (1). The current guidelines recommend that light sedation should be performed in these patients regardless of the sedative choice (1, 2). The Richmond Agitation-Sedation Scale (RASS) score [from −5 (unarousable) to +4 (combative)] is widely used to assess sedation levels (SLs) with a target range of −2 to +1 (3, 4). Several studies have investigated the effects of “light” versus “deep” sedation on clinical outcomes in these patients, based on mean RASS scores, median RASS scores, or the percentage of RASS scores within the target range (5–11). However, in clinical practice, RASS scores can vary widely or fluctuate over time in individual patients (7, 12–14). As such, an average or median score may not accurately represent the SL during a specific period (7, 13). Analysis of the SL variability may provide a novel measure to describe the dynamic nature of changes in the SL over time.

When measured, the RASS score allows practitioners to titrate a patient’s sedatives to a desired SL by observing the patient’s responses to verbal and physical stimulation (3, 15, 16). Therefore, the SL variability may be influenced by the types of sedatives, titration strategy, and patients’ conditions (1, 17, 18). For example, a small SL variability over time may occur in patients whose conditions allow them to express adequate responses to external stimuli and whose SL are well-controlled within the target range. A small SL variability over time may also occur in patients who have repeatedly uncontrolled deep sedation and who have poor organ functions unfavorable for the pharmacokinetics of sedatives (19). Several studies have reported that reduced variability of several physiological responses is associated with poor outcomes in these patients (20–22).

We conducted a retrospective cohort study to investigate the impact of low versus high SL variability as measured during the first 72-h ICU stay on clinical outcomes in ventilated critically ill patients. The SL variability was quantified by calculating the coefficient of variation (CV) using time-series RASS scores for each patient (23). Since the CV is an inappropriate measure when data include both positive and negative values (24), we used transformed RASS score data (exponential RASS score or RASS score plus 6) for the CV measurements.

## 2 Materials and methods

### 2.1 Data source

This retrospective cohort study utilized the Medical Information Mart for Intensive Care IV (MIMIC-IV) database (25), a publicly available resource containing clinical data collected from 2008 to 2019 at the Beth Israel Deaconess Medical Center in Boston, USA. It is one of the few internationally recognized with high-granularity database, specifically the longitudinal RASS scores (up to 18 measurements within 72 h) for calculating the CV for sedation level variability, which is often unavailable in traditional administrative health registries. Ethical approval for the use of data was granted by the Institutional Review Boards at the Massachusetts Institute of Technology. All researchers involved in this study obtained certification (No. 45984821). The study protocol was approved by the Institutional Review Board of Hualien Tzu Chi Hospital (IRB approval number: IRB114-153-C). The informed consent was waived in accordance with institutional policies. The study adhered to the Strengthening the Reporting of Observational Studies in Epidemiology guidelines for observational research in its design, methodology, and reporting.

### 2.2 The original study cohort and design

**Figure 1:**
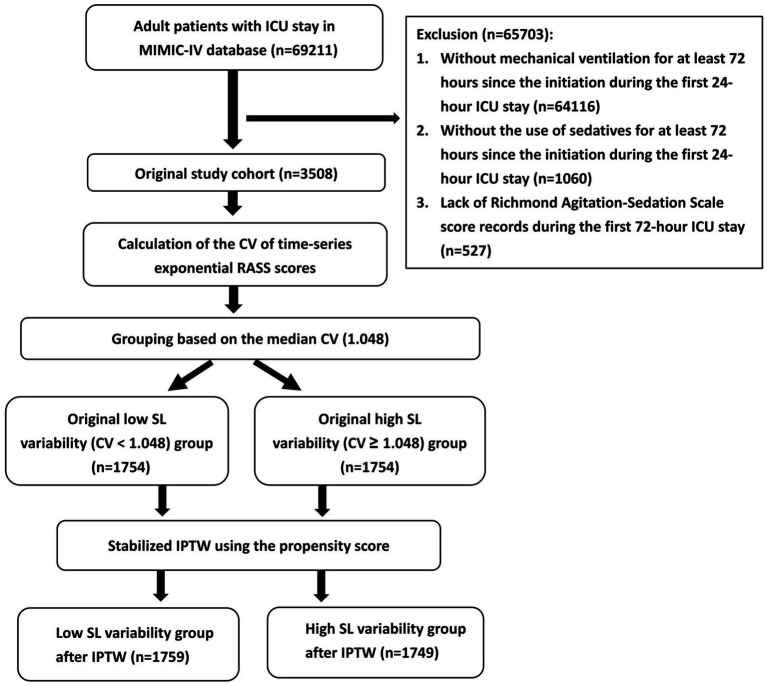
Flowchart of patient disposition. ICU, intensive care unit; MIMIC-IV, Medical Information Mart for Intensive Care IV; CV, coefficient of variation; RASS, Richmond Agitation and Sedation Scale, SL, sedation level; IPTW, inverse probability of treatment weighting.

During 2008 to 2019, we identified patients who required mechanical ventilation and needed intravenous infusion of sedatives as lorazepam, midazolam, propofol, or dexmedetomidine, both of which were initiated within the first 24 h after ICU admission (Figure 1). For patients who were admitted to the ICU more than once during the same study period, only data from the first ICU admission were included. The exclusion criteria were as follows: 1) without mechanical ventilation for at least 72 h since the initiation during the first 24-h ICU stay, 2) without the use of the sedatives for at least 72 h since the initiation during the first 24-h ICU stay, and 3) lack of RASS score records during the first 72-h ICU stay (Figure 1). This design ensured sufficient and consistent sedation exposure and reliable assessment of sedation level variability during the early ICU stay. After exclusion, the original study cohorts were divided into the original low SL (CV < 1.048) and high SL variability (CV ≥ 1.048) groups based on the median of the CV of the SL variability calculated from exponential RASS scores of the original study cohort (Figure 1).

### 2.3 Calculation of the CV serving as the descriptive proxy of SL variability

During the first 72-h ICU stay, each patient had 18 raw RASS score records (one record for every 4 h). The SL variability of each patient in the original study cohort was measured by calculation of the CV of time series RASS scores, which is the ratio of the standard deviation to the mean (23). The raw RASS score data were transformed exponentially (non-linear transformation) or shifted to all positive values by adding 6 to each score (linear transformation) for measurements of the CV (26). This methodology reframes the discrete behavioral observations into a continuous trajectory, allowing for the quantification of SL variability. In this context, the CV serves as a descriptive proxy for the magnitude of fluctuation in a patient’s SL variability.

### 2.4 Covariates

We obtained patients’ demographic data, such as age, sex, and body weight. The patients’ underlying health conditions, including congestive heart failure, chronic obstructive pulmonary disease (COPD), liver cirrhosis, and chronic kidney disease, were also recorded. Additionally, we obtained ICU-related clinical data during the first 24-h ICU stay, including the type and reason of ICU admission, presence of infection, Acute Physiology and Chronic Health Evaluation II (APACHE II) scores, Glasgow Coma Scale (GCS) assessments, the index year of admission, and partial pressure of oxygen/fraction of inspired oxygen (PaO₂/FiO₂) ratios based on arterial blood gas data. Infection was identified based on the presence of antibiotic administration during the first 24-h ICU stay.

We also collected sedation-related information during the first 72-h ICU stay to demonstrate the sedation state with baseline RASS score, median RASS score, and the percentage of RASS scores within target range (−2 to +1) to reflect how often sedation adhered to clinical guidelines. The baseline RASS score was determined as the value recorded closest to the initiation of sedation. The median RASS score for each patient was analyzed using all RASS records collected during the first 72-h ICU stay. Factors like extracorporeal membrane oxygenation (ECMO) use and continuous renal replacement therapy were also included to show if specific high-intensity interventions dictated the sedation patterns.

### 2.5 Outcome measurements and definitions

Outcomes measurements as 28-day mortality was chosen to capture acute ICU-related survival, while 90-day mortality was utilized to assess a long-term perspective on survival, as recommended by recent guidelines (27, 28). In addition, prolonged mechanical ventilation on Day 14 was defined based on the common clinical threshold for difficult weaning, and ventilation-free days on ICU Day 21 was calculated to allow for a balanced assessment of both the duration of ventilation and the competing risk of death (29). For the analysis of prolonged mechanical ventilation on ICU Day 14, individuals who died within the initial 14 days of ICU admission were excluded. For the analysis of ventilation-free days on ICU Day 21, patients who died prior to Day 21 were assigned a value of zero.

### 2.6 Statistical analysis

The Kolmogorov–Smirnov test was used to check the distribution of the continuous variables. Continuous variables were compared using the independent two-sample *t*-test when the data had a normal distribution or the Mann–Whitney *U* test when the data had a non-normal distribution, and are presented as mean ± standard deviation (SD) or median with interquartile ranges (Q1, Q3), respectively. This approach ensures that our statistical inferences are both accurate and appropriate for the underlying data structure.

To minimize selection bias and control for potential confounders, we utilized stabilized inverse probability of treatment weighting (IPTW). First, we calculated propensity scores via a logistic regression model including age, sex, body weight, congestive heart failure, COPD, liver cirrhosis, chronic kidney disease, ICU admission type and reason, APACHE II score, presence of infection, GCS ≤ 8, and the index year. These variables included were selected based on their clinical relevance to both sedation management and mortality risk, for example, demographic data, comorbidities (congestive heart failure, COPD, liver cirrhosis, chronic kidney disease), acute disease severity (APACHE II score, reason of ICU admission), neurological status (GCS), and treatment strategy (ICU type, index year). Stabilized IPTW (30) was then performed to create two balanced pseudo-populations (Figure 1). A standardized mean difference (SMD) < 0.2 indicated a negligible difference between the two study cohorts after IPTW.

Multivariable Cox, multivariable logistic, and ordinary least squares linear regression analyses were used to evaluate the impact of SL variability on outcomes, yielding the adjusted hazard ratio (aHR), adjusted odds ratio (aOR), and adjusted mean difference (aDifference), respectively. The baseline RASS score was adjusted in all of the outcome models. The median RASS score, midazolam use, percentage of RASS scores within target range, and ECMO or renal replacement therapy use during the first 72-h ICU stay were measured after the initial sedatives were given, thus these variables represent as intermediate factors and should not be adjusted in the outcome models. Sensitivity analysis was performed to investigate the impact of the low (first quartile of CV) versus high (fourth quartile of CV) SL variability on the mortality outcomes. Subgroup analyses of the mortality outcomes were performed according to two different percentages (≥60% and <60%) of RASS scores within the target range (11). All statistical analyses were performed using SAS 9.4 for Windows (SAS Institute Inc., Cary, NC, USA). A two-tailed *p*-value <0.05 was considered statistically significant.

## 3 Results

### 3.1 Patient characteristics

During the study period, 69,211 patients were identified. After exclusion, 3,508 patients were included (Figure 1). For each patient, the CV, serving as a descriptive proxy of SL variability, was calculated using 18 exponential RASS scores as measured during the first 72 h of the ICU stay. The median (Q1, Q3) of the CV in this original cohort was 1.048 (0.620, 1.710). Patients were then evenly divided into two original study groups (low versus high SL variability) based on the median of the CV (Figure 1). Subsequently, stabilized IPTW was conducted to produce two pseudo-cohorts: the low (*n* = 1759) and high (*n* = 1749) SL variability groups. IPTW resulted in two well-balanced study groups across all demographic and clinical characteristics (Table 1).

**Table 1:** Demographic and clinical characteristics of the two study groups after stabilized IPTW.

Variable	Low SL variability (*n* = 1759)	High SL variability (*n* = 1749)	SMD
Demographics			
Age, years (SD)	64.24 (16.32)	64.20 (15.94)	0.002
Sex (male), *n* (%)	1,035 (58.27)	1,031 (58.95)	−0.002
Body weight, kg (SD)	83.53 (25.14)	83.46 (24.11)	0.003
Comorbidities			
Congestive heart failure, *n* (%)	482 (27.40)	477 (27.27)	0.002
Chronic obstructive lung disease, *n* (%)	154 (8.75)	153 (8.75)	0.001
Cirrhosis, *n* (%)	146 (8.30)	146 (8.35)	−0.001
Chronic renal disease, *n* (%)	647 (36.78)	642 (36.71)	0.001
Baseline ICU characteristics			
Type of ICU admission, *n* (%)			0.024
CVICU/ CCU	590 (33.54)	584 (33.39)	
MICU	335 (19.04)	334 (19.10)	
MSICU	212 (12.05)	210 (12.01)	
SICU	622 (35.36)	621 (35.51)	
Reason for ICU admission, *n* (%)			0.000
Scheduled surgery	83 (4.72)	81 (4.63)	
Unscheduled surgery	541 (30.76)	537 (30.70)	
Medical	1,135 (64.53)	1,131 (64.67)	
Infection, *n* (%)	190 (10.80)	190 (10.86)	−0.002
APACHE II score (median, Q1–Q3)	29 (24, 33)	28 (25, 33)	0.0020
GCS ≤8, *n* (%)	962 (54.69)	957 (54.72)	−0.001
Index year			0.000
2008–2010	448 (25.47)	445 (25.44)	
2011–2013	460 (26.15)	458 (26.19)	
2014–2016	500 (28.43)	498 (28.47)	
2017–2019	351 (19.95)	348 (19.90)	
PaO_2_/FiO_2_ ratio (median, Q1–Q3)	235.8 (166.9, 334.1)	225.3 (159.1, 309.5)	0.114

Low and high sedation level (SL) variability groups were classified based on the median of the coefficient of variation (CV) of time-series exponential Richmond Agitation and Sedation Scale (RASS) scores measured during the first 72-h ICU stay, and were created by stabilized inverse probability of treatment weighting (IPTW).

SMD, standardized mean difference; SD, standard deviation; ICU, intensive care unit; CVICU, cardiac vascular intensive care unit; CCU, coronary care unit; MICU, medical intensive care unit; SICU, surgical intensive care unit; APACHE, Acute Physiology and Chronic Health Evaluation; GCS, Glasgow Coma Scale; PaO_2_, partial pressure of oxygen; FiO_2_, fraction of inspired oxygen; Q1, Q3, interquartile range. The propensity score model used for IPTW included age, sex, body weight, congestive heart failure, chronic obstructive lung disease, cirrhosis, chronic renal disease, type and reason of ICU admission, infection, APACHE II score, GCS ≤ 8, and index year. An SMD < 0.2 indicates a negligible difference between the two study groups.

### 3.2 Comparisons of the first 72-h ICU characteristics of the two study groups

Table 2 shows the first 72-h ICU characteristics of the two study groups. The baseline RASS score in the low SL variability group was lower compared with that in the high variability group [−4 (−5, −2) versus −3 (−5, −1); *p* < 0.001]. The median RASS scores were −1 (−3, 0) and −1 (−2, 0) in the low and high SL variability groups, respectively (*p* < 0.001). Fewer patients in the low SL variability group received midazolam or lorazepam infusion than the high SL variability group (29.33% versus 34.19%; *p* < 0.002). The low SL variability group had a higher percentage of RASS scores within the target range, compared with the high variability group [75.0% (0.0, 92.9%) versus 57.1% (29.4, 77.8%); *p* = 0.008]. The proportion of patients with the use of ECMO and continuous renal replacement therapy was similar in the two study groups.

**Table 2:** The first 72-h ICU characteristics of the two study groups.

Variable	Low SL variability (*n* = 1759)	High SL variability (*n* = 1749)	*p*-value
Baseline RASS score, median (Q1, Q3)	−4 (−5, −2)	−3 (−5, −1)	**<0.001**
Median RASS score, median (Q1, Q3)	−1 (−3, 0)	−1 (−2, 0)	**<0.001**
Midazolam or lorazepam continuous infusion, *n* (%)	516 (29.33)	598 (34.19)	**0.002**
Percentage of RASS scores within the target range, % (median, Q1–Q3)	75.0 (0.0, 92.9)	57.1 (29.4, 77.8)	**0.008**
ECMO support, *n* (%)	6 (0.34)	5 (0.29)	0.745
Continuous renal replacement therapy, *n* (%)	193 (10.97)	188 (10.75)	0.820

The baseline RASS score was defined as the RASS score closest to the time of initial sedative administration. The median RASS score was calculated using all RASS records collected during the first 72-h ICU stay. The target range was defined as RASS score −2 to +1. *p*-value with bold font indicates statistically significant.

ICU, intensive care unit; SL, sedation level; RASS, Richmond Agitation and Sedation Scale; Q1, Q3, interquartile range. ECMO, extracorporeal membrane oxygenation.

### 3.3 Impact of the low versus high SL variability on ICU outcomes

**Figure 2:**
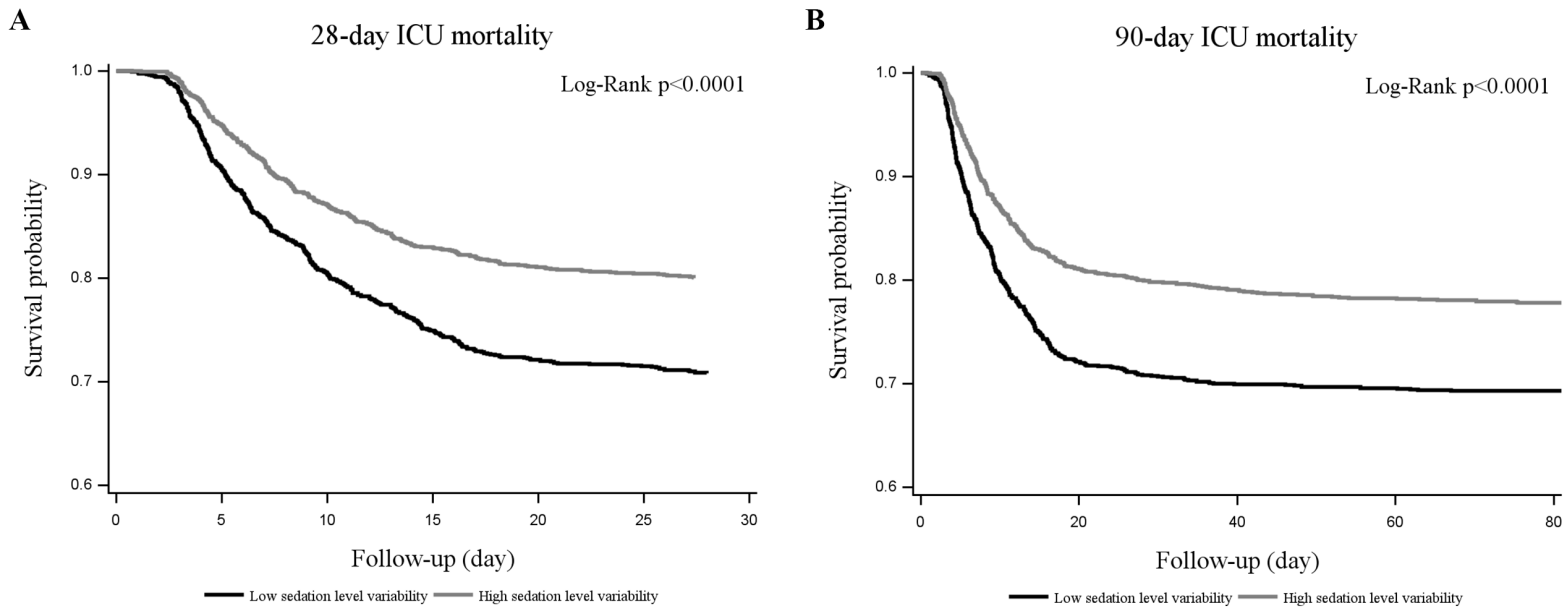
Kaplan–Meier curves for 28-day **(A)** and 90-day **(B)** ICU mortality. Patients in the low sedation level (SL) variability group showed a significant increase in risk of death at 28 days or 90 days (Log-Rank test, *p* < 0.0001), compared with patients in the high SL variability group. ICU, intensive care unit.

The low SL variability group had a higher risk of 28-day [aHR = 1.57 (1.36, 1.81); *p* < 0.001] and 90-day ICU mortality [aHR = 1.51 (1.32, 1.72); *p* < 0.001], compared with the high variability group (Table 3). However, there was no significant impact of the low SL variability on prolonged mechanical ventilation on ICU Day 14 [aOR = 1.20 (0.97, 1.48); *p* = 0.103] and ventilation-free days on ICU Day 21 [aDifference = −0.45 (−0.95, 0.05) days; *p* = 0.077] (Table 3). The Kaplan–Meier curve analysis revealed that the cumulative incidence rate of death in the low SL variability group was significantly higher (Figure 2; Log-Rank test, *p* < 0.0001) than that in the high SL variability group over the follow-up periods.

**Table 3:** The impact of the low versus high sedation level variability on various ICU outcomes.

28-day ICU mortality, *n* (%)	Low SL variability (*n* = 1759)	High SL variability (*n* = 1749)	HR (95% CI)	aHR (95% CI)	*p*-value
	513 (29.16)	351 (20.07)	1.55 (1.36, 1.78)	1.57 (1.36, 1.81)	**<0.001**
90-day ICU mortality, *n* (%)	Low SL variability (*n* = 1759)	High SL variability (*n* = 1749)	HR (95% CI)	aHR (95% CI)	*p*-value
	543 (30.87)	390 (22.30)	1.48 (1.30, 1.69)	1.51 (1.32, 1.72)	**<0.001**
Prolonged mechanical ventilation on ICU Day 14, *n* (%)	Low SL variability (*n* = 1,291)	High SL variability (*n* = 1,424)	OR (95% CI)	aOR (95% CI)	*p*-value
	215 (16.65)	208 (14.61)	1.17 (0.95, 1.44)	1.20 (0.97, 1.48)	0.103
Ventilation-free days on ICU Day 21, median (Q1, Q3)	Low SL variability (*n* = 1759)	High SL variability (*n* = 1749)	Difference (95% CI)	aDifference (95% CI)	*p*-value
	12.26 (0, 16.73)	13.38 (0.37, 16.38)	−0.34 (−0.82, 0.14)	−0.45 (−0.95, 0.05)	0.077

The patients who died within 14 days were excluded from the analysis of prolonged mechanical ventilation on ICU Day 14. The patients who died before day 21 were regarded as having zero days when analyzing the ventilation-free days on day 21. The baseline RASS score was adjusted in all of the regression models. The high SL variability group was used as the reference group. *p*-value with bold font indicates statistically significant.

ICU, intensive care unit; SL, sedation level; Q1, Q3, interquartile range; HR, hazard ratio; aHR, adjusted hazard ratio; OR, odds ratio; aOR, adjusted odds ratio; Difference, mean difference; aDifference, adjusted mean difference; CI, confidence interval.

We performed additional analysis using the CV calculated from the transformed RASS scores, which were shifted to all positive values by adding 6. IPTW resulted in two well-balanced study groups across all demographic and clinical characteristics (Supplementary Table S1). Consistently, the low SL variability group had a higher risk of 28-day ICU mortality [aHR = 1.20 (1.05, 1.38); *p* = 0.007] and 90-day ICU mortality [aHR = 1.20 (1.05, 1.36); *p* = 0.007], compared with the high variability group (Supplementary Table S2).

### 3.4 Sensitivity and subgroup analyses

In the sensitivity analysis (Table 4), patients in the low and high SL variability groups had the first and fourth quartiles, respectively, of the CV of exponential RASS scores. Similarly, the low SL variability group had a higher risk of 28-day [aHR = 1.86 (1.54, 2.23); *p* < 0.001] and 90-day ICU mortality [aHR = 1.78 (1.49, 2.13); *p* < 0.001], compared with the high variability group. Additional stratified analysis (Table 5) revealed that, in the subgroup with < 60% of RASS scores within the target range, but not in the subgroup with ≥ 60%, the low SL variability was associated with a higher risk of 28-day ICU mortality [aHR = 2.60 (2.16, 3.13); *p* < 0.001] and 90-day ICU mortality [aHR = 2.50 (2.09, 2.99); *p* < 0.001].

**Table 4:** Sensitivity analysis of the impact of the low (first quartile of CV) versus high (fourth quartile of CV) sedation level variability on the ICU mortality.

ICU 28-day mortality, *n* (%)	Low SL variability (*n* = 876)	High SL variability (*n* = 857)	HR (95% CI)	aHR (95% CI)	*p*-value
	329 (37.56)	189 (22.05)	1.92 (1.61, 2.29)	1.86 (1.54, 2.23)	**<0.001**
ICU 90-day mortality, *n* (%)	Low SL variability (*n* = 876)	High SL variability (*n* = 857)	HR (95% CI)	aHR (95% CI)	*p*-value
	343 (39.16)	207 (24.15)	1.84 (1.55, 2.18)	1.78 (1.49, 2.13)	**<0.001**

Patients in the low and high sedation level (SL) variability groups had the first and fourth quartiles, respectively, of the coefficient of variation (CV) of exponential RASS scores measured during the first 72-h ICU stay.

ICU, intensive care unit; HR, hazard ratio; aHR, adjusted hazard ratio; CI, confidence interval. The baseline RASS score was adjusted in the regression model. The high SL variability group was used as the reference group. *p*-value with bold font indicates statistically significant.

**Table 5:** Subgroup analysis regarding percentage of RASS scores within target range of the impact of the low versus high sedation level variability on ICU mortality.

28-day ICU mortality, (event/total)	Low SL variability	High SL variability	HR (95% CI)	aHR (95% CI)	*p*-value
≥60% of RASS scores within the target range	126/1042	118/832	0.85 (0.66, 1.09)	0.90 (0.69, 1.17)	0.424
<60% of RASS scores within the target range	387/717	233/917	2.62 (2.23, 3.09)	2.60 (2.16, 3.13)	**<0.001**
90-day ICU mortality (event/total)	Low SL variability	High SL variability	HR (95% CI)	aHR (95% CI)	*p*-value
≥60% of RASS scores within the target range	139/1042	134/832	0.83 (0.65, 1.05)	0.88 (0.69, 1.13)	0.305
<60% of RASS scores within the target range	404/717	256/917	2.53 (2.16, 2.96)	2.50 (2.09, 2.99)	**<0.001**

The target range was defined as RASS score −2 to +1. The baseline RASS score was adjusted in the analysis of the subgroup with ≥ 60% of RASS scores within the target range. The baseline RASS score, type of ICU admission, and Glasgow Coma Scale ≤ 8 were adjusted in the analysis of the subgroup with < 60% of RASS scores within the target range. The high SL variability group was used as the reference group. *p*-value with bold font indicates statistically significant.

ICU, intensive care unit; SL, sedation level; RASS, Richmond Agitation and Sedation Scale; HR, hazard ratio; aHR, adjusted hazard ratio; CI, confidence interval.

## 4 Discussion

In this investigation, the two study groups after IPTW had similar clinical characteristics. Notably, as compared to patients in the high SL variability group, the low SL variability group showed a significant increase in risk of 28- or 90-day mortality. In contrast, these two study groups had similar outcomes regarding prolonged mechanical ventilation on ICU Day 14 and ventilation-free days on ICU Day 21. Our findings regarding mortality are consistent when analyses were performed using exponential RASS scores (main data) or RASS scores shifted to all positive values (Supplementary material). The demographic and clinical characteristics of two IPTW cohorts remained well-balanced (Supplementary Table S1), and the association between low SL variability and increased mortality remained statistically significant (Supplementary Table S2). This consistency across different calculation methods reinforces our conclusion that SLV serves as a robust descriptive marker of sedation patterns during the early ICU phase. The sensitivity analysis further revealed that these mortality risks increased among patients whose CV was in the first quartile compared with those with the CV in the fourth quartile significantly, suggesting the robustness of our findings. Subgroup analysis showed that the negative impact of the low SL variability on 28- or 90-day mortality was evident only in the subgroup with < 60% of RASS scores within the target range, but not in the subgroup with ≥ 60%.

The guidelines recommend using light sedation, with a target range of RASS −2 to +1, in the critically ill whenever possible (1–4). In clinical practice, it is not always feasible to predict how an individual patient will respond. In our study, only 53.4% of patients had ≥ 60% of RASS scores within the target range during the first 72-h ICU stay. As such, RASS scores can vary widely or fluctuate over time in an individual patient (7, 12–14). Our results indeed show that ICU patients exhibited a wide range of SL variability. Only one previous study reported that a greater variability in SL may increase the incidence of delirium in ICU patients (31). However, that investigation used the CV calculated from raw RASS scores, which is not valid (24).

Several randomized controlled trials and prospective studies (5, 7, 8), as well as retrospective observational studies (6, 11) and meta-analyses (9, 32, 33) have investigated the effects of “light” versus “deep” sedation on clinical outcomes, although the findings have been inconsistent. One recent meta-analysis reported that inhaled sedation has advantages over intravenous sedation in terms of awakening time, extubation time, and ICU length of stay (34). Most previous studies have defined “light” and “deep” sedation based on mean RASS scores (7, 8), median RASS scores, or the percentage of RASS scores within the target range (5, 6, 11). However, these measures cannot describe the dynamic nature of changes in the SL over time (7), and cannot accurately represent the SL during a specific period (7, 13). It is possible that two groups of patients with the same values of these measures may exhibit vastly different SL variability over time. In this study, the median RASS score calculated from the first 72-h ICU stay in the low SL variability group, was numerically similar to that in the high SL variability group. Thus, the characteristics of the low and high SL variability groups are irrelevant to “light” or “deep” sedation.

The exact reason why reduced SL variability had a negative impact on mortality remains unclear. Essentially, the RASS scoring system categorizes various patients’ responses to verbal and physical stimulation (3, 15, 16). Therefore, the SL variability may be influenced by the types of sedatives and titration strategy (1, 17). However, several patient factors should also be taken into consideration. For example, patients with poor physiological function in responding to external stimuli may exhibit lower degrees of SL variation. A recent study reported that impaired neurological and neurophysiological lower brainstem responses were associated with mortality in deeply sedated patients (35). Additionally, patients with poor blood flow and organ function may exhibit lower degrees of SL variation due to unfavorable alterations in the pharmacokinetics of sedatives, which affect absorption, distribution, metabolism, and excretion (19). It has been suggested that preserved variability of the physiological responses is frequently a sign of better health condition in ICU patients (20).

We additionally observed that the negative impact of low SL variability on mortality was not seen in the subgroup with ≥ 60% of RASS scores within the target range. Patients in this subgroup are assumed to have favorable physiological conditions responding to external stimuli. In these patients, the low SL variability may simply reflect the situation that their SL were well controlled within the target levels, thereby not affecting the risk of mortality. On the other hand, the negative impact of low SL variability on mortality was found in the subgroup with < 60% of RASS scores within the target range. Judging from the median of RASS scores, these patients may have repeatedly uncontrolled deep sedation most of the time during the first 72-h ICU stay. As such, these patients may suffer from the known detrimental effects of “deep” sedation on clinical outcomes (5–7). Also, the low SL variability in this subgroup suggests that these patients may have poor physiological reserve, and their SL may tend to remain deep over time. It is known that patients who maintain < 60% of RASS scores within the target range have unfavorable ICU outcomes (11). Reduced SL variability in this context may not represent an entirely novel construct, but rather a complementary metric that captures the lack of behavioral fluctuation often seen in patients receiving intensive or deep sedation. Our findings underscore the importance of further identifying patients with low SL variability within this subgroup, as they are at an increased risk of mortality compared to those with high SL variability.

In this study, we found there were no significant impacts of the low SL variability on prolonged mechanical ventilation on ICU Day 14 and ventilation-free days on ICU Day 21, in contrast to the mortality findings. As such, this discrepancy may suggest that the physiological impact of low SL variability might be more closely linked to a lack of physiological reserve or neurological reactivity rather than isolated respiratory recovery, therefore was not associated with outcomes related to ventilator use in ICU.

A key strength of our study is the use of a large-scale biomedical database that integrates comprehensive real-world clinical information from diverse ICU settings. The second strength is that we were able to collect the RASS scores immediately after sedation initiation for each patient, which is often omitted in prospective studies due to delay of data collection related to screening and enrollment. This granular approach captures the true dynamic nature of sedation level in critically ill patients. The third strength is the robust confounding control by employing IPTW, we were able to balance the most relevant baseline covariates between the low and high SL variability groups. This approach may reduce the selection bias and approximate the balance of a randomized trial in a real-world setting. However, several limitations need to be considered. First, this work is a retrospective study subject to several inherited biases between the two study cohorts. We believe that these biases could be minimized mostly via stabilized IPTW. However, unmeasured confounders may still account for our observed results. Refinement of the propensity score model to include indications for sedation will be essential to mitigate the bias associated with treatment intensity.” Second, we acknowledge the mathematical limitations of the use of CV with ordinal clinical scales and offer it as a proxy for SL variability. Given the ordinal nature of the RASS score, the use of CV in this study is presented as an exploratory descriptive tool to summarize the magnitude of behavioral fluctuations. Third, our study cohort included patients admitted to large medical centers in the U.S., which limits the generalizability of these findings. Future studies involving multi-center data from diverse geographical regions are warranted to validate these findings across different clinical practices.

## 5 Conclusion

Our study identifies that reduced SL variability in the early ICU phase is descriptively associated with increased 28- and 90-day mortality in the ventilated critically ill. Patients with < 60% of RASS scores within the target range are vulnerable to this negative impact of reduced SL variability. Rather than acting as a modifiable prognostic tool, low SL variability may serve as a quantified marker of a stagnant sedation trajectory. These findings underscore the importance of monitoring dynamic sedation patterns, although further prospective research is required to determine if these patterns can be intentionally modified to improve clinical outcomes.

## Data Availability

The original contributions presented in the study are included in the article/Supplementary material, further inquiries can be directed to the corresponding author.
