# Supplementary material for: Sedation level variability as an indicator for mortality in mechanically ventilated critically ill patients: a propensity score-weighted cohort study
Source: Front Med (Lausanne). 2026 Jan 16;13:1755288. doi: 10.3389/fmed.2026.1755288 (PMC12855395; doi:10.3389/fmed.2026.1755288)
Supplement: Supplementary file 1 [file Table_1.DOCX]

Supplementary Material: 1755288

# Supplementary Tables (2 tables) uploaded separately on submission

**TABLE S1** Demographic and clinical characteristics of the two study groups after stabilized IPTW.

|  | **Low SL variability (n=1770)** | **High SL variability (n=1741)** | **SMD** |
| --- | --- | --- | --- |
| **Demographics** |  |  |  |
| Age, years (SD) | 63.82 (16.60) | 63.90 (15.96) | -0.005 |
| Sex (male), n (%) | 1045 (59.04) | 1020 (58.59) | 0.009 |
| Body weight, kg (SD) | 83.82 (24.74) | 83.78 (24.51) | 0.002 |
| **Comorbidities** |  |  |  |
| Congestive heart failure, n (%) | 471 (26.61) | 462 (26.54) | 0.002 |
| Chronic obstructive lung disease, n (%) | 151 (8.53) | 148 (8.50) | 0.001 |
| Cirrhosis, n (%) | 151 (8.53) | 151 (8.67) | -0.005 |
| Chronic renal disease, n (%) | 652 (36.84) | 635 (36.47) | 0.008 |
| **Baseline ICU characteristics** |  |  |  |
| Type of ICU admission, n (%) |  |  | 0.028 |
| CVICU/ CCU | 588 (33.22) | 571 (32.80) |  |
| MICU | 345 (19.49) | 337 (19.36) |  |
| MSICU | 214 (12.09) | 210 (12.06) |  |
| SICU | 623 (35.20) | 623 (35.78) |  |
| Reason for ICU admission, n (%) |  |  | 0.049 |
| Scheduled surgery | 80 (4.52) | 75 (4.31) |  |
| Unscheduled surgery | 541 (30.56) | 536 (30.79) |  |
| Medical | 1149 (64.92) | 1130 (64.91) |  |
| Infection, n (%) | 187 (10.56) | 187 (10.74) | -0.007 |
| APACHE II score (median, Q1-Q3) | 29.0 (24.0, 33.0) | 28.0 (24.0, 33.0) | 0.009 |
| GCS ≤8, n (%) | 966 (54.58) | 949 (54.51) | 0.001 |
| Index year |  |  | 0.029 |
| 2008 - 2010 | 442 (24.97) | 437 (25.10) |  |
| 2011 - 2013 | 474 (26.78) | 464 (26.65) |  |
| 2014 - 2016 | 505 (28.53) | 495 (28.43) |  |
| 2017 - 2019 | 349 (19.72) | 345 (19.82) |  |
| PaO2/FiO2 ratio (median, Q1-Q3) | 235.1 (162.0, 330.2) | 226.0 (159.8, 316.4) | 0.068 |

Low and high sedation level (SL) variability groups were classified based on the median of the coefficient of variation (CV) of time-series transformed RASS scores (shifted the RASS scores to all positive values by plus 6 to each score) measured during the first 72-hour ICU stay, and were created by stabilized inverse probability of treatment weighting (IPTW).

SMD, standardized mean difference; SD, standard deviation; ICU, intensive care unit; CVICU, cardiac vascular intensive care unit; CCU, coronary care unit; MICU, medical intensive care unit; SICU, surgical intensive care unit; APACHE, Acute Physiology and Chronic Health Evaluation; GCS, Glasgow Coma Scale; PaO_2,_ partial pressure of oxygen; FiO_2_, fraction of inspired oxygen; Q1, Q3, interquartile range. The propensity score model used for IPTW included age, sex, body weight, congestive heart failure, chronic obstructive lung disease, cirrhosis, chronic renal disease, type and reason of ICU admission, infection, APACHE II score, GCS ≤ 8, and index year. An SMD < 0.2 indicates a negligible difference between the two study groups.

**TABLE S2** The impact of the low versus high sedation level variability on ICU mortality.

| **28-day ICU mortality, n (%)** | **Low SL variability (n=1770)** | **High SL variability (n=1741)** | **HR (95% CI)** | **aHR  (95% CI)** | **P-value** |
| --- | --- | --- | --- | --- | --- |
|  | 490 (27.68) | 428 (24.58) | 1.15 (1.01, 1.31) | 1.20 (1.05, 1.38) | **0.007** |
| **90-day ICU mortality, n (%)** | **Low SL variability (n=1770)** | **High SL variability (n=1741)** | **HR (95% CI)** | **aHR  (95% CI)** | **P-value** |
|  | 528 (29.83) | 463 (26.59) | 1.15 (1.02, 1.30) | 1.20 (1.05, 1.36) | **0.007** |

The baseline Richmond Agitation and Sedation Scale score was adjusted in the regression model. The high SL variability group was used as the reference group. P-value with bold font indicates statistically significant.

ICU, intensive care unit; SL, sedation level; Q1, Q3, interquartile range; HR, hazard ratio; aHR, adjusted hazard ratio; CI, confidence interval.

**
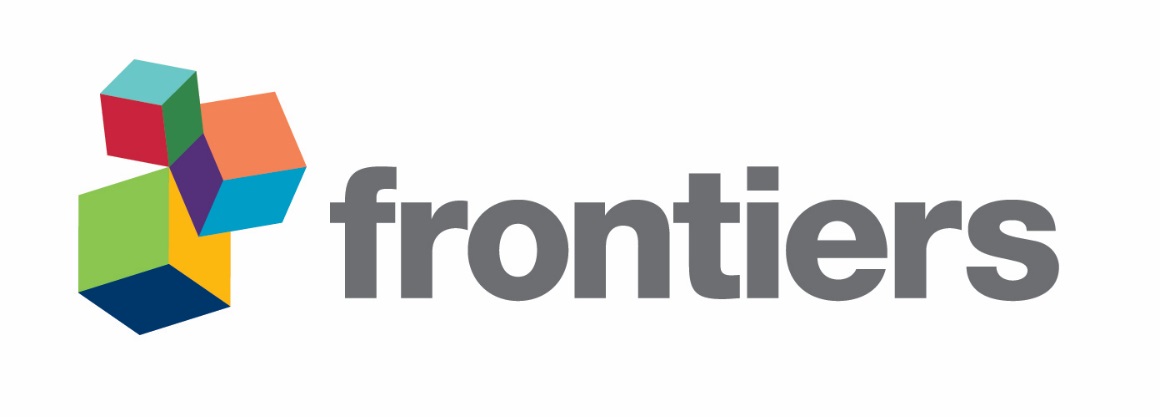
**
